# Presence of Post-traumatic Limps in an Adult Population Diagnosed on a “Catwalk Video”: A Comparative Study Between Healthcare and Nonhealthcare Individuals

**DOI:** 10.7759/cureus.46369

**Published:** 2023-10-02

**Authors:** Andrew Robinson, Lauryn Boggs, Maiko G Ebersole, Rahul Vaidya

**Affiliations:** 1 Orthopedic Surgery, Wayne State University Detroit Medical Center, Detroit, USA; 2 Family Medicine, University of Kansas Health System, Kansas City, USA

**Keywords:** walking patterns, gait abnormalities, gait, crowdsourcing, medical screening, two-dimensional video, limp, gait disorders, post traumatic limp, gait analysis

## Abstract

Background

Walking is an everyday activity but also complex in nature. Gait disorders have the potential to drastically affect an individual's quality of life and their ability to be independent. The causes of gait disorders are numerous. To identify abnormal gait, clinicians utilize gait analysis. The aim of this study is to assess how well individuals can identify limps in postoperative traumatized individuals with lower extremity deformities.

Methods

Participants observed a video compiled of individuals with various gait abnormalities and severities of limps. In the video, there were nine abnormal gait presentations, four obvious limps, and five subtle limps, while the other 10 gait presentations were normal gaits. Classifications for gait presentations were assigned by the research team. Participants assigned a classification to each limp case presented in the video on a survey. The participants were separated into two groups: those with healthcare experience and lay individuals. A Mann-Whitney U-test was used to compare healthcare experience and lay individuals' ability to identify limps correctly. In addition, the observers were evaluated on their ability to perform a screening diagnosis of a limp.

Results

A total of 100 participants were included in the study, 46 with healthcare experience and 54 individuals without. All tests, identification of limp and subtle limp, using the Mann-Whitney U-test yielded non-significant differences between healthcare and nonhealthcare experience. Overall lowest correctness between both groups came when attempting to identify subtle limp (healthcare = 57.39%, nonhealthcare = 56.67%) while the highest correctness yield was when identifying limp (healthcare = 96.74%, nonhealthcare = 95.37%). Analysis of the observers’ ability to perform a screening diagnosis of limp provided close to gold standard results (sensitivity = 96.0%, specificity = 98.7%, positive predictive value = 99.2%, negative predictive value = 98.4%).

Conclusion

This study showed that nonhealthcare individuals can accurately perform gait analysis from a video, particularly in identifying the presence of a limp, to a similar extent as individuals with healthcare experience. The implementation of two-dimensional catwalk videos taken from a smartphone is beneficial due to accessibility and cost-effectiveness. It also suggested that limp diagnosis can be done as a screening test, using individuals as the screener.

## Introduction

Walking is an everyday routine task, but is also complex in nature, possessing inherent intricacies. Normal walking is a rhythmic and symmetrical process that occurs effortlessly [1-4]. It involves all levels of the nervous system, many parts of the musculoskeletal system, and even the cardiovascular system. The unique way that an individual walks is known as their gait. A person’s gait is heavily influenced by their age, personality, and mood [5]. Gait disorders have the potential to drastically affect an individual's quality of life and their ability to be independent. The causes of gait disorders are numerous and can be classified as orthopedic conditions, neuromuscular conditions, and other causes such as psychological disorders [1,5,6]. 

A limp is described as an abnormal pattern of walking where the movements of one or both legs differ from the norm [1,5-7]. In a gait cycle, each step is divided into two primary phases: the stance and the swing phase. The stance phase consists of the time the foot is in contact with the ground while the swing phase consists of the time the foot is in the air. Each of the phases can be divided up into different subsections. During the stance phase, the foot bears partial or all of the body’s weight. It is divided into three sections: heel-strike, mid-stance, and push-off. The swing phase occurs in the air, it begins as the toe leaves the ground after push-off and ends as the heel strikes the ground. It is separated into three sections: acceleration, swing-through, and deceleration [1,6,8,9]. During gait analysis, each of these steps must be carefully assessed to determine any abnormalities indicative of a limp. Identifying the cause of a limp can be a complex task [6-10].

One of the goals after lower extremity trauma is to return to a normal gait. In orthopedic surgical procedures, the identification and classification of limp can help decide on what surgery is necessary [11] and what rehabilitation is necessary after orthopedic procedures. The presence of a limp signifies an ongoing deficit that needs correction or rehabilitation [12]. A limp is either present or absent. We wanted to see how well the general population could identify a limp in patients rehabilitating from lower extremity trauma. 

This study aims to assess how well individuals can identify limps in post-operation traumatized individuals. We also hypothesized that most individuals could discern when an individual has a limp without inherently understanding the necessary steps to analyze gait.

## Materials and methods

An IRB-approved prospective study was performed on 100 individuals, 46 with healthcare experience and 54 lay individuals, who were selected from an orthopedic clinic in Detroit, MI at random and areas around the US as the video and survey were shared digitally. Participants who participated virtually were selected based on whether they would have healthcare experience and willingness to partake in the study. These individuals were contacted, questioned, and informed about the purpose of the study. Not all of the virtual participants had healthcare experience, but the research member attempted to replicate a diverse sampling with respect to age and gender. They were age 18+ with no specific occupation requirements. They were questioned with regard to name, age, occupation, and whether they had any experience in healthcare.

Participants were included in one of the groups (healthcare experience or lay individual) based on their responses to the survey question about whether they have any healthcare experience. In addition, the occupation of the individual was taken into account when determining which group to assign them to. Participants who stated they had little healthcare experience were included in the lay individual group. Participants were excluded if occupation or healthcare experience were not included in their responses. 

The patients selected for inclusion in the videography with various limp cases were all individuals with lower extremity fractures, at our orthopedic trauma clinic, were fully weight-bearing, and had already started rehabilitation. Table 1 shows the fracture diagnosis, limp classification, and post-operative recovery duration for each limp case.

**Table 1:** Limp Cases: Classification, Fracture Diagnosis, and Recovery Duration

Limp Case	Limp Classification	Fracture Diagnosis	Post-operative Recovery Duration
Case #1	Obvious Limp	Close comminuted intertrochanteric fracture of the proximal end of right femur	12 months
Case #2	Obvious Limp	Left distal femur nonunion	26 months
Case #3	Subtle Limp	Closed fracture of posterior malleolus of left tibia; Closed fracture of posterior malleolus of left tibia; Closed left pilon fracture	17 months
Case #4	Obvious Limp	Left tibia fracture complicated by osteomyelitis	39 months
Case #5	Obvious Limp	Close bicondylar fracture of left tibial plateau; Closed dislocation of left knee	19 months
Case #6	Subtle Limp	Right tibia fracture complicated by osteomyelitis	26 months
Case #7	Subtle Limp	Closed fracture of shaft of left tibia and fibula; Closed fracture of shaft of left tibia and fibula	2 months
Case #8	Subtle Limp	Right hip fracture and dislocation	28 months
Case #9	Subtle Limp	Closed displaced fracture of right femoral neck; Closed displaced fracture of right femoral neck	2 months

As it is difficult to show the same subject walking to a large group of individuals, videos were made of these patients walking away and then toward the camera (in this case an iPhone) which is commonly done to assess gait or change in gait (catwalk video) (Video 1). 

**Video 1:** Catwalk Video With Various Limp Cases

**Figure 1:**
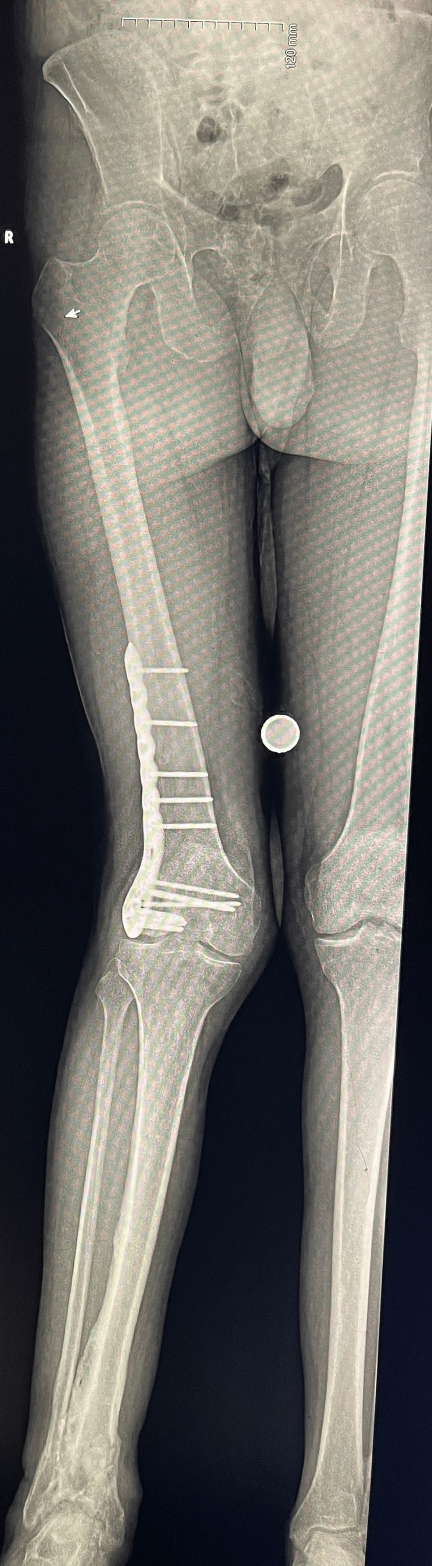
Patient X-Ray

The etiology of the patient’s limps differed; the gait abnormalities were varied. In the video, there were nine abnormal gait cases, four obvious limps, and five subtle limps. The patients were identified by the senior authors and labeled. Major and minor classification terms were used to ease understanding for lay individuals. However, the terms are more accurately described as limp (major limp) and subtle limp (minor limp) and will be used throughout the remainder of the paper. Abnormal limp cases were defined as major or obvious limps if the research team could observe and diagnose a limp for a particular gait case within five seconds of the presentation. If more observation time was required to diagnose with a limp, the gait case would be labeled as a minor or subtle limp. Provided below is an x-ray of a patient in the video with an obvious limp representation (Figure 1). The cause for this individual’s limp is deformity.

Within the mix of the compiled video cases, a group of 10 normal gait cases, serving as controls, were dispersed throughout. These individuals were also recruited in the clinic, but they were either family members of patients or patients with upper extremity deformities that had no effect on their ability to walk. These individuals had no signs of a limp and were screened for conditions that could cause a limp, ensuring suitability as normal gait presentations. The normal gait group was also filmed in the same clinic hallways so that they weren’t easily distinguishable.

Survey

A survey was created to record participant responses concerning whether the individuals in the video displayed a limp. Google Forms was used to create and administer the survey. Participants who participated in the clinic were administered the survey by a research member on a computer. Whereas, virtual participants received a link to the survey. The survey consisted of 22 questions: name, occupation, a question about whether or not they had any experience in healthcare, and 19 questions about whether the cases in the video displayed a limp (see Appendices). The survey and video were shared via a digital link. 

Participants surveyed in the clinic used two devices to complete the survey. Participants who completed the elsewhere were asked to use two devices for convenience. This way they could view the video and record responses simultaneously. However, under the circumstance where two devices weren’t available, participants could use one device but had to pause between each presented gait case when marking responses. Participants were permitted to rewind the video and were given ample time to record their responses. The survey was administered by the same member of the research team in every case.

Respondents of the survey were given the option to remain anonymous and/or not disclose any information concerning their occupation or experience in health care. However, if a participant omitted their healthcare experience, their results were excluded as the results compare those with healthcare experience to those without. For the question concerning whether the individuals in the video displayed a limp, there were 3 options to choose from: no limp, minor limp, and major limp.

## Results

A total of 100 participants were included in this study, 46 individuals with healthcare experience and 54 individuals without. Individuals within the healthcare experience group included a wide range of professionals: nurses, physicians, physical therapists, and medical students. The inclusion within the healthcare experience group was self-determined by participants. None had ever had any training specific to gait assessment or analysis. The results will be explored as if the observers were conducting a screening test for possible gait abnormality. In addition, the comparison of participant groups was observed in multiple scenarios: identification of limp and subtle limp; identification of all limp cases regardless of classification based on correctness; and misidentification of no limp or normal, subtle limp, and limp based on occurrences.

Screening test

All respondents were evaluated on their ability to perform a screening diagnosis of a limp. The criteria for a true positive result was that the observer identified a limp as either such or a subtle limp. The criteria for a false positive result was identifying a normal gait presentation as a limp. The total screening number for this sample was 1400; 400 limp cases and 1000 normal gait cases. Each participant, a total of 100, screened 4 limp cases and 10 normal gait cases. From this screening test, the sensitivity was 96.0% and the specificity was 98.7%. The positive predictive value was 99.2% and the negative predictive value was 98.4%. This shows that the screening test is very close to that of the gold standard and thus can be utilized effectively to diagnose or screen for a limp in individuals. In this case, it was not an actual screening test that was evaluated, but rather individuals’ ability to screen.

Identification of limp

The Mann-Whitney U-test was used to compare those with healthcare experience to lay individuals in correctly identifying limp. A participant correctly identified a limp when identified as such or a subtle limp. On average, participants with healthcare experience correctly identified limp 96.74 percent of the time with a standard deviation of 8.51 percent. Lay participants correctly identified a limp 95.37 percent on average with a standard deviation of 13.80 percent. The Mann-Whitney U-test showed a non-significant difference in the identification of limp between the two groups (p-value = 0.953).

Identification of subtle limp

The Mann-Whitney U-test was used to compare those with healthcare experience to those without in correctly identifying subtle limps. On average, participants with healthcare experience correctly identified subtle limps 57.39 percent of the time with a standard deviation of 25.160 percent. Lay participants correctly identified subtle limps 56.67 percent on average with a standard deviation of 24.495 percent. The Mann-Whitney U-test showed a non-significant difference in the identification of subtle limp between the two groups (p-value = 0.963). 

Identification of limp cases regardless of classification 

The Mann-Whitney U-test was used to compare the group’s ability to correctly identify limp cases, 9 total, regardless of classification. On average, participants with healthcare experience correctly identified limp cases, subtle limp or limp, 78.50 percent of the time with a standard deviation of 15.784 percent. Lay participants correctly identified a limp case 77.98 percent on average with a standard deviation of 16.648 percent. The Mann-Whitney U-test showed a non-significant difference in the identification of limp regardless of classification between the two groups (p-value= 0.983). 

Number of misidentifications of subtle limp

The Mann-Whitney U-test was used to compare the two group's misidentification of a subtle limp. On average, participants with healthcare experience misidentified 3.67 subtle limps with a standard deviation of 1.777. Lay participants misidentified 3.24 subtle limps with a standard deviation of 1.873. The Mann-Whitney U-test showed a non-significant difference in the misidentification of subtle limp between the two groups (p-value= 0.238). 

Number of misidentifications of limp

The Mann-Whitney U-test was used to compare the number of misidentifications of a limp. On average, participants with healthcare experience misidentified 0.33 limps with a standard deviation of 0.560. Lay participants misidentified 0.41 limps with a standard deviation of 0.659. The Mann-Whitney U-test showed a non-significant difference in the misidentification of limp between the two groups (p-value = 0.575).

Number of misidentifications of limp absence

The Mann-Whitney U-test was used to compare the number of misidentifications of an absence of a limp. On average, participants with healthcare experience misidentified 1.96 limp absence with a standard deviation of 1.398. Lay participants misidentified 1.98 limp absence with a standard deviation of 1.498. The Mann-Whitney U-test showed a non-significant difference in the misidentification of limp absence between the two groups (p-value = 0.946).

## Discussion

The presence of gait abnormality or “limp” after fracture treatment is a common problem and the goal of rehabilitation is to normalize the gait pattern [4]. We performed this study to assess how well the general population can identify post-traumatic limp. The study group consisted of 100 random people who watched videos of patients walking down and back (could repeat it) and were asked if the patient had a limp (gait anomaly). The survey showed that the study group could identify limp on this screening test video with a sensitivity of 96.0% and specificity was 98.7%. It also showed that there was no significant difference between people of healthcare background and no healthcare background to perform this task. The Mann-Whitney U-test showed a non-significant difference in the identification of limp between the two groups (p-value = 0.953). In other words, anyone can spot a limp regardless of background as it is an “Easy Task” even on a video and can identify subjects without a limp as well. 

An explanation for the ability of the population to identify limp is the historical existence of ableism [13]. It is embedded within a society to identify those who are physically impaired. Although individuals may not understand why someone is limping, it can be easily distinguished because people have a good understanding of what is considered “normal”. This doesn’t just apply to limps as people can discern when someone isn’t from a particular community because they don’t fit the characteristics that define the community. Ableism is discrimination or social prejudice against people who are disabled or perceived to be such. In humans, the presence of a limp is often associated with disability. Ableism promotes the idea that disabled individuals are inferior. While attitudes have changed, the identification of disabled individuals has not subsided. While viewing all individuals as equal is the expected social norm, it isn’t possible for society yet to completely ignore a disability without identifying it [13]

However, both healthcare and nonhealthcare experienced people had difficulty assessing subtle limp. Both groups correctly identified subtle limp cases on average approximately 57% of the time. Due to low accuracy, it cannot be inferred that either group has the ability to access subtle limp. Having healthcare experience has no effect on the participants' abilities to assess subtle limp. The use of screening with nonhealthcare individuals has been used in multiple settings. Sabri et al. demonstrated this for visual impairment screening of lay individuals with a 75% success rate after a 40-hour training program [14]. Perhaps with some type of extra gait training subtle limp could be identified correctly.

Research exists displaying the use of two-dimensional video-based analysis of the human gait. Sternum et al compared two-dimensional video-based analysis to the commonly used three-dimensional motion capture and found only slight differences, with 0.02 absolute error for temporal gait parameters [15]. This study had software perform the gait analysis. The study revealed that two-dimensional videos taken from a smartphone can be used and reviewed by an individual to determine the presence of a limp. The three-dimensional motion capture technology is expensive, immobile, and requires some degree of expertise.

Despite producing results that supported the hypothesis, there are limitations to the study. One of the limitations is the limp case sample size. It would have been better for statistical analysis had there been more than four or five cases of each limp classification. However, getting individuals to partake in the study would have been more of a challenge as it would require much more time if the preferred data set was at least 10 cases of each limp classification. Another limitation of the study could be the self-definition of the healthcare experience. Strict criteria for inclusion in the healthcare group or limiting to healthcare professionals specializing in human locomotion may have been appropriate. 

Future studies addressing similar topics should take these limitations into consideration. A future study may show that it is not possible to determine the cause of a limp from video analysis alone. This future study would be practical because it would suggest that while clinical visits are still necessary, one could query a lay individual about possible gait abnormalities before scheduling appointments. Additionally, the study could provide a clear methodology for a clinical assessment of limp etiology and treatment.

## Conclusions

In conclusion, this study has shown and supports that nonhealthcare individuals can accurately perform gait analysis from a video, particularly in identifying the presence of a limp. The results suggest that medical screenings, such as gait analysis, can be completed with almost equal accuracy to that of trained healthcare professionals. However, the results do not suggest that nonhealthcare workers can determine a cause associated with each limp case. In addition, the results suggest that limp diagnosis can be done as a screening test using lay individuals as the screener. 

This study also showed the implementation of two-dimensional videos taken from a smartphone and reviewed by individuals to determine the presence of a limp is a viable approach to gait analysis. This approach is beneficial as it is more accessible and cost-effective compared to the expensive, technically complex, and immobile three-dimensional motion capture technology. Lastly, this study suggests the possibility and capability of using crowdsourcing for the medical screening of limps as the video containing the limps was shared via the internet.

## Appendices

Participant's Survey

Question list:

- Name:

- Occupation:

- Do you have any experience in healthcare?

- Does case #1 display a limp? Answer options: no limp, minor limp, or major limp

- Does case #2 display a limp? Answer options: no limp, minor limp, or major limp

- Does case #3 display a limp? Answer options: no limp, minor limp, or major limp

- Does case #4 display a limp? Answer options: no limp, minor limp, or major limp

- Does case #5 display a limp? Answer options: no limp, minor limp, or major limp

- Does case #6 display a limp? Answer options: no limp, minor limp, or major limp

- Does case #7 display a limp? Answer options: no limp, minor limp, or major limp

- Does case #8 display a limp? Answer options: no limp, minor limp, or major limp

- Does case #9 display a limp? Answer options: no limp, minor limp, or major limp

- Does case #10 display a limp? Answer options: no limp, minor limp, or major limp

- Does case #11 display a limp? Answer options: no limp, minor limp, or major limp

- Does case #12 display a limp? Answer options: no limp, minor limp, or major limp

- Does case #13 display a limp? Answer options: no limp, minor limp, or major limp

- Does case #14 display a limp? Answer options: no limp, minor limp, or major limp

- Does case #15 display a limp? Answer options: no limp, minor limp, or major limp

- Does case #16 display a limp? Answer options: no limp, minor limp, or major limp

- Does case #17 display a limp? Answer options: no limp, minor limp, or major limp

- Does case #18 display a limp? Answer options: no limp, minor limp, or major limp

- Does case #19 display a limp? Answer options: no limp, minor limp, or major limp
